# Comprehensive rheological characterization of polynucleotide/BDDE-crosslinked hyaluronic acid blend for intra-articular viscosupplementation

**DOI:** 10.1038/s41598-025-22080-5

**Published:** 2025-10-31

**Authors:** Hwanwoo Choi, Young Seok Song, Dongwhan Suh

**Affiliations:** 1 Department of Fiber Convergence Materials Engineering, Dankook University, Seoul, Gyeonggi Do 16890 Republic of Korea; 2 Joint Replacement Center, Department of Orthopedics, Bumin Hospital, 389, Gonghang- daero, Gangseo-gu, Seoul, Republic of Korea

**Keywords:** Rheology, Viscosupplement, Osteoarthritis, Hyaluronic acid, Polynucleotide, Preclinical research, Gels and hydrogels, Biomedical materials, Rheology

## Abstract

Viscosupplementation is a widely used treatment for osteoarthritis, aiming to restore the viscoelastic properties of synovial fluid and improve joint function. While hyaluronic acid (HA), BDDE-crosslinked hyaluronic acid (BDDE-HA), and polynucleotide (PN) have been studied individually, the rheological properties of their mixtures remain insufficiently explored. This study investigates the viscoelastic characteristics of PN/HA and PN/BDDE-HA blends through rheological evaluations, including simple shear tests, oscillatory shear tests, and 3-interval thixotropy tests. Results indicate that BDDE-HA maintains high viscosity over a range of shear rates, whereas PN exhibits viscosity loss at high shear rates. PN/HA exhibits synergistic structural recovery properties, consistent with clinical findings suggesting improved efficacy compared to individual components. PN/BDDE-HA exhibits improved viscoelastic behavior but prolonged structural recovery. These findings highlight the importance of comprehensive rheological analysis in understanding viscosupplement formulations and their potential clinical implications. This study provides fundamental data to support future clinical research on PN/BDDE-HA as a novel viscosupplement.

## Introduction

The primary role of the knee joint synovial fluid (SF) is known as a lubricant^1,2^, composed of lubricin, albumin, hyaluronic acid, and phospholipid. The representative lubrication mechanism of SF, in which viscosity plays a critical role, includes hydrodynamic and elastohydrodynamic lubrication for friction reduction, as well as squeeze-film and boundary lubrication for impact absorption^3–5^.

Osteoarthritis is characterized by a constellation of features, among which a notable viscosity reduction in SF is prominent^3^. This decrement is partially attributed to inflammatory processes within the synovial tissue ^6–8^. Viscosupplementation is a therapeutic intervention involving the injection of a viscoelastic fluid into the joint to alleviate symptoms by supplementing SF. Previous research showed that viscosupplement (VS) has chondroprotective effect by facilitating joint lubrication, mitigating shear stress, and reducing inflammation^9–11^.

Hyaluronic acid (HA), crosslinked HA, and polynucleotide (PN) are VS whose efficacy has been validated through numerous clinical studies^12–18^. HA, a naturally occurring polysaccharide, is known for its viscoelastic properties and high water retention capacity. It is a prevalent ingredient in VS; nevertheless, its rapid degradation in vivo imposes limitations on its long-term efficacy^19^. Crosslinking of HA is a strategy to enhance its performance. 1,4-butanediol diglycidyl ether (BDDE) is commonly used as a crosslinking agent, and HA crosslinked with BDDE (BDDE-HA) demonstrates enhanced viscosity and mechanical stability to degradation. This effect is attributed to the chemical linkage of hyaluronic acid molecules, which results in the formation of a three-dimensional network structure. This structural modification restricts the molecular movement and significantly increases the overall rigidity of the structure. PN is a DNA-derived polymer that has garnered attention as a substitute for HA. PN exhibits viscoelasticity and anti-inflammatory characteristics analogous to HA^20,21^.

Clinical comparison studies among these VS have shown varied results. BDDE-HA clearly offers advantages in terms of injection frequency compared to HA, but further research is needed to determine its superiority in pain relief^22–24^. There are studies suggesting that PN is clinically comparable to HA or even superior to both HA and BDDE-HA^25,26^. A noteworthy study was conducted in which a clinical trial was performed using a mixture of PN and HA (PN/HA)^27,28^. These studies demonstrated that PN/HA exhibited a significant improvement in pain relief compared to PN and HA alone.

The objective of this study is to address the paucity of rheological analysis on PN/HA, as no prior studies have systematically investigated their viscoelastic properties. Furthermore, a rheological analysis of a mixture of PN and BDDE-HA (PN/BDDE-HA) has not been performed nor studied in clinical applications. Given the growing interest in VS, understanding the relationship between rheological properties and clinical outcomes is essential. This study also aims to contribute to the body of knowledge by conducting a preliminary rheological investigation of PN/BDDE-HA, with the objective of providing insights that could support future clinical research on its potential therapeutic benefits.

## Methods

### Sample preparation

Two widely available commercial VS; PN (Conjuran, Pharma Research, Korea, 20 mg/ml, molecular weight = 1000 kDa) and BDDE-HA (Synovian, LG Chem, Korea, 20 mg/ml, molecular weight > 10,000 kDa) were used in rheological tests. The HA (Sigma-Aldrich, USA, molecular weight = 15,000–18,000 kDa) solution was prepared at a concentration of 10 mg/ml using deionized water as the solvent. The concentration of HA was established on the basis of concentrations that have been reported to be clinically safe and efficacious^29^. PN/HA and PN/BDDE-HA were prepared by blending using a planetary centrifugal mixer (AR-100, Thinky, Japan) for 5 min.

### Rheological measurements

The rheological properties of the samples were examined using a rheometer (MCR-302, Anton Paar, Austria) equipped with parallel plates geometry (a diameter of 25 mm and a 1 mm gap). All experiments were carried out at a temperature of 31.4 ℃, the intra-articular temperature of knee joint at a stationary state^30^. All rheological measurements were repeated five times with independent sample loadings. The results demonstrated negligible variation, thereby confirming high reproducibility.

The simple shear tests of the samples were conducted at shear rates ranging from 0.1 to 100 1/s. The amplitude sweep tests were conducted by adjusting the shear strain in the range of 0.01–100%, under the condition of 0.1 rad/s. The frequency sweep tests of the samples were performed in the range of 0.1–100 rad/s at a 0.5% strain within the linear viscoelastic region (LVR) determined from the amplitude sweep tests. 3iTT procedure employed an oscillation-rotation-oscillation method. In the first interval, the samples were subjected to the strain within the LVR of 0.5% for a duration of 120 s. In the second interval, a rotation was applied at the shear rate of 100 1/s, which was selected to induce pronounced structural deformation, for 10 s. The third interval involved measuring the samples at the strain of 0.5% for 300 s. The frequency was set to 1 rad/s.

## Results

### Simple shear test

**Fig. 1:**
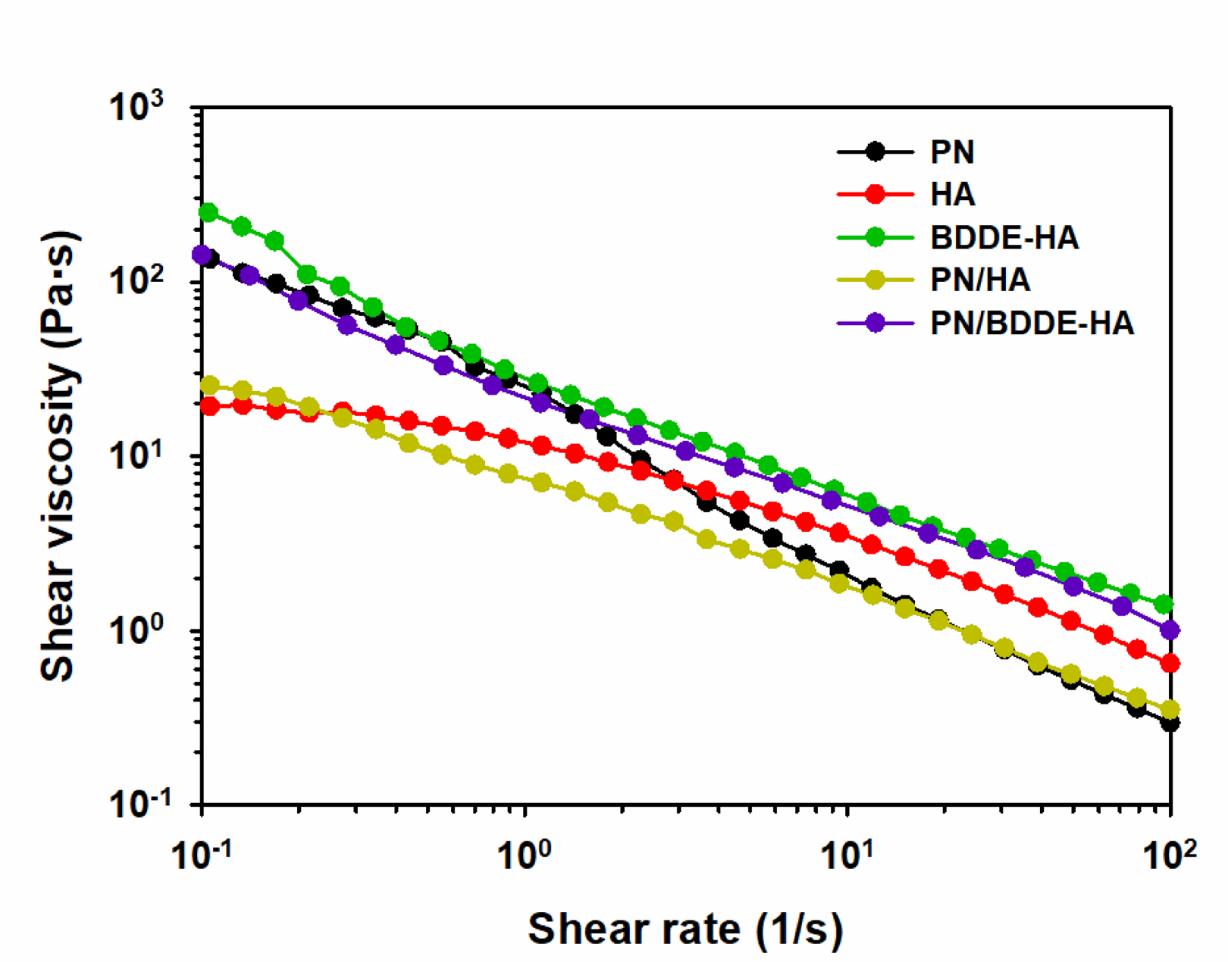
Shear viscosity as a function of shear rate for PN, HA, BDDE-HA, PN/HA, and PN/BDDE-HA. Measurements were conducted at a temperature of 31.4 ℃ within a shear rate range of 0.1–100 1/s.

The simple shear tests were conducted to evaluate a shear stability of VS samples. In Fig. 1, BDDE-HA exhibited consistently high viscosity across the entire range of shear rates, indicative of its notable resistance to flow and suggesting mechanical stability provided by the cross-linked network. Conversely, the viscosity of PN decreased with the applied shear rate, suggesting its vulnerability to structural failure. This behavior demonstrates that the internal network of PN is susceptible to mechanical failure under dynamic conditions. HA showed the opposite trend. It had the lowest viscosity at low shear rates, but maintained its viscosity more effectively at high shear rates. This suggests that HA has shear-damping properties with moderate resistance to high shear degradation, which may be due to its entangled structure that is effective at rapid deformation.

Among the blends, PN/BDDE-HA exhibited a viscosity profile analogous to that of BDDE-HA, thereby suggesting that the cross-linked HA component exerts a substantial influence on the flow resistance. This finding indicates that the presence of BDDE-HA mitigates the high-shear viscosity loss typically observed in PN. In contrast, the PN/HA blend exhibited the lowest viscosity among all samples, with a noticeable viscosity reduction in the medium shear rate range. This synergistic viscosity reduction suggests limited intermolecular reinforcement between PN and HA, which may be due to the absence of relatively strong interpolymer interactions or entanglements. This is reasonable given that both PN and HA are negatively charged polyelectrolytes, which makes spontaneous chemical bonding between them improbable.

The shear viscosity data not only reflect the compositional differences, but also provide insight into the fundamental structural stability of each material under flow. These results suggest that BDDE-HA demonstrates the capacity to preserve its structural integrity across a broad range of shear rates. In contrast, PN and PN-containing blends exhibit diminished mechanical strength when not reinforced with cross-linked HA.

The power-law model is an intuitive model for describing the flow behavior of fluids, and it can be expressed as follows:

1 $$\eta = K{\dot{\gamma}}^{n-1}$$

where $\eta$ is the shear viscosity ($\text{P}\text{a}\cdot \text{s}$), $\dot{\gamma}$ is the shear rate ($1/s$), $K$ is the flow consistency index ($\text{Pa}\cdot {\text{s}}^{n}$), and $n$ is the flow behavior index (-). The magnitude of $K$ represents the resistance of the fluid to flow, while $n$ characterizes the shear-dependent behavior: shear-thinning when $n<1,$ Newtonian when $n=1,$ and shear-thickening when $n>1.$ The power-law parameters obtained through a simple regression analysis were used to quantitatively describe the data in Fig. 1, and the results are summarized in Table 1.

**Table 1:** Power-law parameters and coefficient of determination (${\text{R}}^{2}$) of various VS samples.

	$K({\text{P}\text{a}\cdot\text{s}}^{n}$)	$n$ (-)	${\text{R}}^{2}$ (-)
PN	20.7	0.045	0.988
HA	14.2	0.357	0.991
BDDE-HA	28.2	0.343	0.999
PN/HA	8.26	0.319	0.997
PN/BDDE-HA	22.6	0.348	0.999

### Oscillatory shear test

**Fig. 2:**
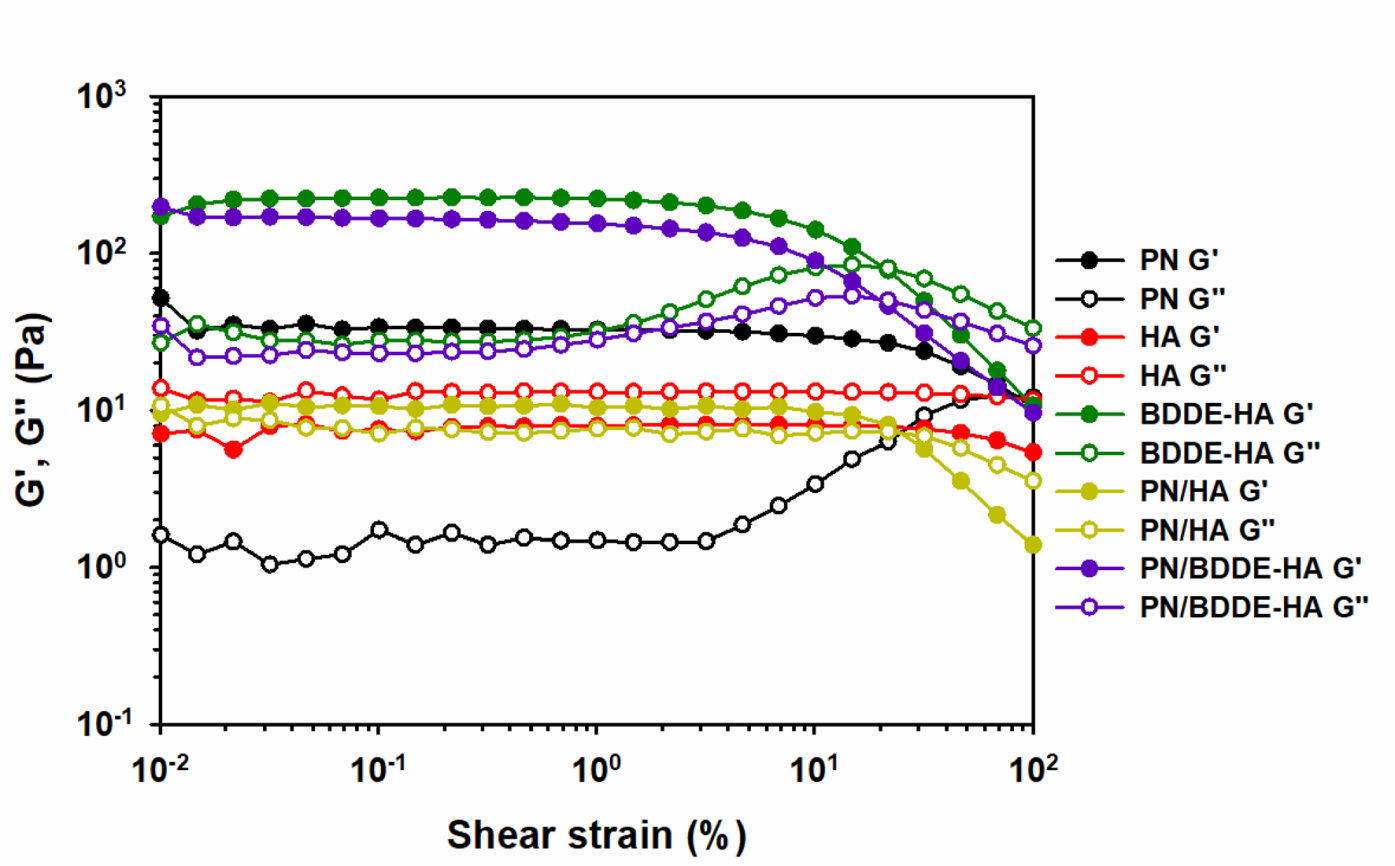
Amplitude sweep results showing storage modulus (${G}^{{\prime}}$) and loss modulus ($\:{G}^{{\prime}{\prime}}$) as a function of strain for PN, HA, BDDE-HA, PN/HA, and PN/BDDE-HA. Measurements were conducted at a temperature of 31.4 ℃ and a frequency of 1 rad/s within a strain range of 0.01–100%.

The amplitude sweep tests were conducted to assess the structural stability of the samples against shear. The storage modulus (${G}^{{\prime}}$) and loss modulus (${G}^{{\prime}{\prime}}$) are indicative of the elastic and viscous components of viscoelasticity, respectively. In Fig. 2, the ${G}^{{\prime}}$ values within LVR exhibited trends that were consistent with those observed in the low shear rate region of the simple shear test. With the exception of HA, all samples demonstrated elasticity-dominant behavior, as evidenced by ${G}^{{\prime}}$ exceeding ${G}^{{\prime}{\prime}},$ suggesting the presence of well-developed internal network structures. As the strain increased, structural breakdown occurred first in BDDE-HA and PN/BDDE-HA, followed sequentially by PN, PN/HA, and HA. A notable observation was the most pronounced reduction in ${G}^{{\prime}}$ at high strain for PN/HA, in contrast to the behavior of PN/BDDE-HA, whose ${G}^{{\prime}}$ values closely followed those of PN and BDDE-HA. This finding suggests a deficiency in mechanical synergy within the PN/HA blend, while the preserved elasticity in PN/BDDE-HA may be ascribed to the robust crosslinking facilitated by BDDE-HA.

The amplitude sweep results indicate that the structural stability under deformation is highly dependent on crosslinking and polymer compatibility. The blends with BDDE-HA and PN exhibit superior mechanical properties, while PN/HA does not contribute to the reinforcement of the elastic network at the high shear strain.

**Fig. 3:**
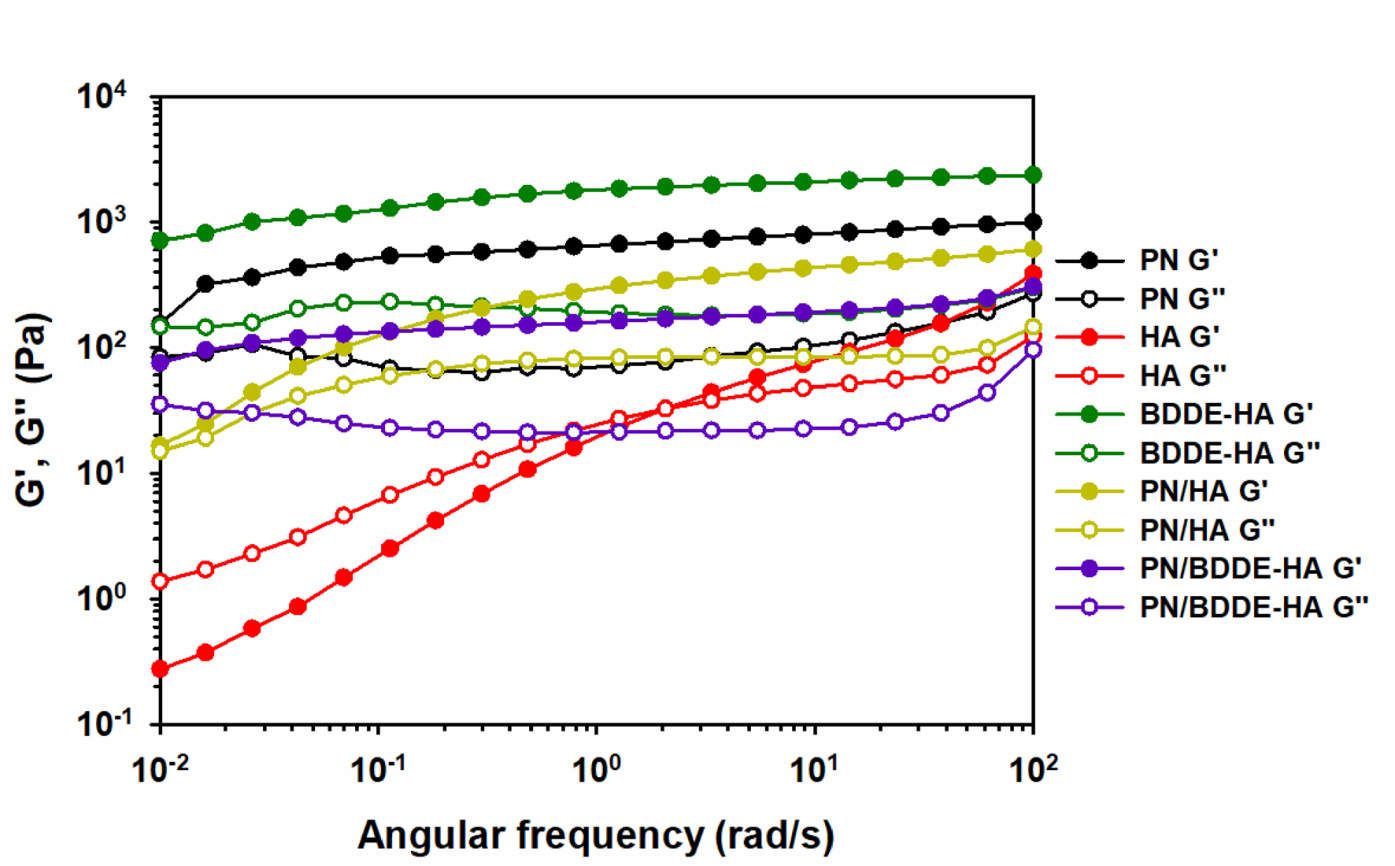
Frequency sweep results showing storage modulus (${G}^{{\prime}}$) and loss modulus (${G}^{{\prime}{\prime}}$) as a function of frequency for PN, HA, BDDE-HA, PN/HA, and PN/BDDE-HA. Measurements were conducted at a temperature of 31.4 ℃ and a strain of 0.5% within a frequency range of 0.01–100%.

Figure 3 represents the results of the frequency sweep test to analyze the time-dependent viscoelastic behavior of a material. In the frequency range that was measured, all samples except HA exhibited relatively stable ${G}^{{\prime}}$ and ${G}^{{\prime}{\prime}}$ values. This finding suggests that the samples have a well-organized internal structure that resists time-dependent deformation. The elastic dominance was observed to persist in BDDE-HA, PN, and PN/BDDE-HA, suggesting that their network structures exhibit resistance to long-term or slow oscillatory deformation. In contrast, HA exhibited frequency-sensitive behavior. At high frequencies, the structure remained stable. At low frequencies, however, both ${G}^{{\prime}}$ and ${G}^{{\prime}{\prime}}$ decreased, indicating that there is limited viscoelastic recovery at slow deformations. This observation suggests that the internal structure of HA exhibits low cohesion, which renders it poorly resistant to long-term deformation.

The PN/HA blend followed a transitional pattern, showing intermediate behavior between PN and HA. ${G}^{{\prime}}$ values were consistently lower in the PN/HA blend compared to PN alone, though this decrease was less pronounced in the low-frequency range, suggesting the potential for partial reinforcement. Conversely, the PN/BDDE-HA blend exhibited a stable ${G}^{{\prime}},$ indicative of the response of BDDE-HA and reinforcing the structural dominance of the cross-linked HA component.

The frequency sweep results demonstrate that PN, BDDE-HA, and PN/BDDE-HA exhibit stable viscoelastic properties across a wide frequency range, indicating low sensitivity to deformation timescales. In contrast, HA and PN/HA exhibited increased sensitivity to the strain time scale. This finding suggests that HA may be disadvantageous in terms of long-term mechanical stability.

### 3-interval Thixotropy test

3-interval thixotropy test (3iTT) was conducted to evaluate the structural recovery of the samples after exposure to high shear strain. ${G}^{{\prime}}$ was measured during interval 1 and interval 3, at an amplitude of 0.5%, in order to simulate a resting state. Interval 2 applied high shear to induce structural breakdown. Structural deformation occurs when polymer chains in solution are stretched and aligned under applied shear stress, thereby disrupting their equilibrium random coil conformation. The structural deformation rate was quantified using following equation:

2 $$Deformation\:rate=\left(\frac{{G}_{f}^{{\prime}}-{G}_{i}^{{\prime}}}{{G}_{i}^{{\prime}}}\right) \times 100$$

where ${G}_{i}^{{\prime}}$ is the initial ${G}^{{\prime}}$ of interval 3 and ${G}_{f}^{{\prime}}$ is the final ${G}^{{\prime}}$ value of interval 1. Subsequent to the cessation of shear, the polymer chains undergo a gradual relaxation back to their original equilibrium state. In 3iTT, the recovery time was defined as the duration required for ${G}^{{\prime}}$ in interval 3 to reach the final ${G}^{{\prime}}$ value of interval 1.

**Fig. 4:**
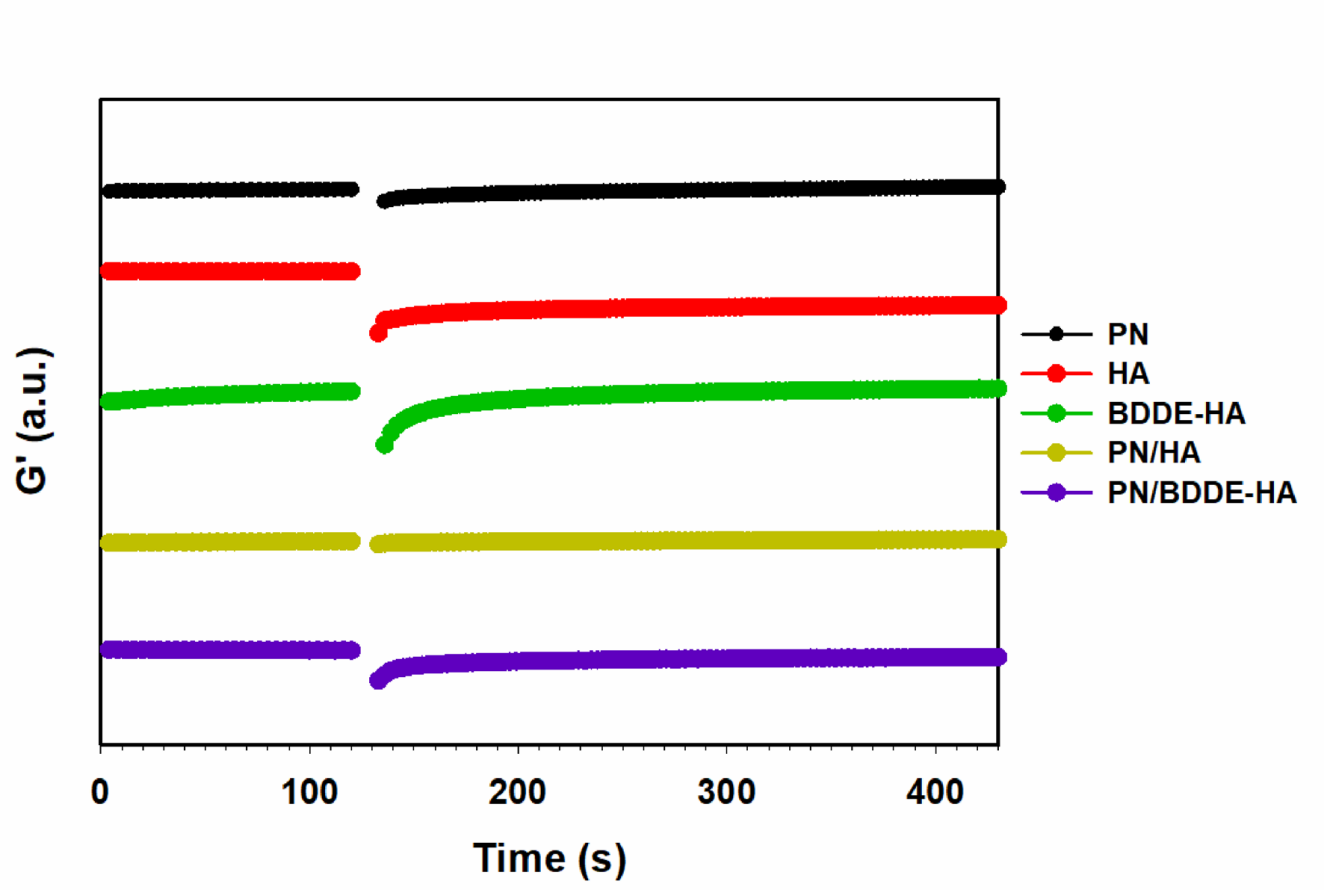
3-interval thixotropy test results for PN, HA, BDDE-HA, PN/HA, and PN/BDDE-HA. The first interval was conducted at a strain of 0.5% (within LVR) for 120 s, followed by the second interval at a shear rate of 100 1/s for 10 s to induce structural deformation. The third interval entailed monitoring recovery at a strain of 0.5% for 300 s. All tests were conducted at a temperature of 31.4 ℃ with oscillation intervals set at a frequency of 0.1 rad/s.

In Fig. 4, PN demonstrated the lowest structural strain at 30.99% and exhibited a recovery time of 108 s to the original ${G}^{{\prime}}$ level, indicating moderate resistance to fracture and relatively well-preserved internal structure. On the other hand, HA showed the highest structural strain (71.78%) and failed to recover within the measurement time, demonstrating poor elastic recovery ability under repeated stress. BDDE-HA exhibited a structural strain of 56.49%, indicating its vulnerability to high shear forces due to its rigid cross-linked network. However, it exhibited a rapid recovery within 57 s, thereby demonstrating the reversibility of the viscoelastic structure. This rapid recovery can be attributed to the elasticity of the cross-linked matrix, which can be reformed after disruption.

PN/HA exhibited a 33.06% strain reduction in comparison with HA alone, and attained complete structural recovery within a mere 12 s. This observation indicates that the blending with PN enhances the structural stability and thixotropic behavior of HA, which is possible due to the formation of a temporary reinforcing network. However, despite the moderate strain of 31.90%, PN/BDDE-HA did not fully recover within the observed time interval. Nevertheless, a gradual increase in ${G}^{{\prime}}$ was observed after shear, suggesting a slow but gradual structural recovery. The deformation rates and recovery times of the samples are summarized in Table 2.

**Table 2:** Deformation rate and recovery time obtained from 3-interval Thixotropy test for PN, HA, BDDE-HA, PN/HA, and PN/BDDE-HA. Deformation rate was calculated from the reduction in storage modulus (${G}^{{\prime}}$) after high-shear application, and recovery time was defined as the duration required to reach the last G′ of the first interval. N/A indicates that full recovery was not achieved within the measurement period.

	Deformation rate (%)	Recovery time (s)
PN	30.99	108
HA	71.78	N/A
BDDE-HA	56.49	57
PN/HA	33.06	12
PN/BDDE-HA	31.90	N/A

## Discussion

This study provides a comprehensive rheological comparison of PN, HA, BDDE-HA, PN/HA, and PN/BDDE-HA. The structural response of these materials under conditions relevant to intra-articular loading was investigated through a series of evaluations, including shear, oscillatory, and thixotropic tests.

PN demonstrated a substantial reduction in viscosity under high shear. However, PN exhibited relatively slight deformation and gradual structural recovery, indicating stable network retention and resilience under repeated loading. HA showed relatively low viscosity and delayed deformation recovery after high shear, indicating inadequate resistance to both rapid and gradual mechanical stresses. Conversely, BDDE-HA demonstrated a substantial enhancement in properties when compared to HA, a result of its chemically crosslinked network. The superior viscosity and structural recovery characteristics support the clinical findings that BDDE-HA can maintain therapeutic efficacy even with reduced injection frequency compared to non-crosslinked HA formulations^23,24^.

In comparison with HA alone, PN/HA exhibited superior structural recovery and diminished deformation. Although the blend demonstrated diminished viscoelasticity under oscillatory shear in contrast to BDDE-HA-based formulations, the rapid recovery observed in 3iTT suggests the potential for clinical benefits in scenarios demanding short-term mechanical resilience. This observation is consistent with the findings of previous studies that indicated that PN/HA demonstrates superior clinical efficacy in comparison to the separate utilization of PN or HA^27,31.^

PN/BDDE-HA exhibited viscoelastic properties, primarily governed by the BDDE-HA component, and was characterized by high viscosity and elasticity-dominant behavior. Although full recovery was not achieved within the 3iTT measurement period, a continuous increase in ${G}^{{\prime}}$ was observed, indicating progressive structural restoration. This finding indicates that PN/BDDE-HA may exhibit superior mechanical stability under sustained or dynamic loading conditions in comparison to PN/HA. Specifically, PN/BDDE-HA demonstrates promise in clinical scenarios where durability is prioritized over rapid recovery. From a mechanical standpoint, higher shear viscosity and elastic dominance are expected to sustain load-bearing lubrication and damping under low-to-moderate shear, typical of daily activities. This has the potential to prolong functional residence and symptomatic relief. Conversely, the slower structural recovery implies that following high-shear situations (e.g., squatting, stair ascent/descent), the network requires a longer re-formation time, during which transient softening may occur. The relatively slow recovery behavior could be addressed through future compositional optimization, thereby enabling a balance between the advantages of crosslinking and enhanced network reversibility.

In previous rheological studies of SF, characteristic power-law parameters have been reported. Normal SF typically exhibits $K>0.03$ and $n<0.85,$ whereas inflammatory SF is generally characterized by $K<0.01$ and $n>0.85$^32^. This indicates that normal SF possesses increased viscosity and demonstrates a more pronounced shear-thinning response in comparison to inflammatory SF. The data presented in Table 1 demonstrate that all samples tested fall within the rheological range reported for normal synovial fluid. Given that the power-law parameters were obtained within the shear range exhibiting power-law behavior, the trend of $K$ does not perfectly correspond with that of Fig. 1. Nevertheless, the values of $n$ provide meaningful insights into the sensitivity of the samples to shear-thinning. In particular, in contrast to the other samples that exhibit relatively similar $n$ values, PN displays a remarkably pronounced shear-thinning behavior. This finding suggests that the internal network of PN undergoes a rapid breakdown under shear. The susceptibility of PN to shear may offer a rationale for its blending with materials possessing relatively higher $n$ values, thereby enhancing its rheological robustness.

Injectability is a clinically significant parameter for intra-articular formulations. It is important to note that both PN (Conjuran) and BDDE-HA (Synovian) are already commercially available and clinically used for intra-articular applications. This strongly indicates their inherent injectability. In the present study, the flow curves (Fig. 1) demonstrated that the viscosities of the PN/HA and PN/BDDE-HA formulations fall within the range defined by the individual PN and BDDE-HA components. Given the established correlation between injectability and viscosity^33,34^, particularly within the context of shear-thinning phenomena during injection, these findings imply that the mixed formulations are anticipated to adhere to clinically acceptable injectability parameters. Additional factors, such as needle gauge, syringe design, and local shear conditions, must also be considered when assessing the efficacy of a given injection technique. While rheological data provide strong indirect evidence, dedicated injectability measurements would be valuable for future studies to directly validate the clinical handling performance of these formulations.

While the study demonstrated promising enhancements in viscoelastic properties, further research is necessary to account for physiological factors such as enzymatic exposure and interactions with the synovial membrane, which may influence in vivo behavior. Furthermore, the current study exclusively evaluated a 1:1 mixing ratio. In polymer blend systems, the physical properties generally adhere to a rule of mixture, whereby intermediate compositions can be estimated based on the behaviors of the individual components. Accordingly, a 1:1 ratio was selected in this study as a representative midpoint composition, providing a practical reference to assess synergistic or antagonistic rheological effects. While a more comprehensive evaluation of multiple ratios may offer additional insights, the 1:1 mixture serves as a balanced benchmark within the scope of this study. Nevertheless, it is imperative for future research to explore a more extensive compositional spectrum to identify rheologically optimized blends.

The present study is the first to provide a rheological characterization of the PN/BDDE-HA blend. Additionally, this study offers a preliminary investigation into the rheological properties of PN/BDDE-HA, providing foundational data that may inform subsequent investigations into its clinical applicability and formulation optimization.

## Data Availability

The datasets analyzed during the current study are available from the corresponding author on reasonable request.

## References

[1] Hui, A. Y., McCarty, W. J., Masuda, K., Firestein, G. S. & Sah, R. L. A systems biology approach to synovial joint lubrication in health, injury, and disease. *Wiley Interdiscipl. Rev. Syst. Biol. Med.* **4**, 15–37 (2012). 10.1002/wsbm.157 PMC3593048 21826801

[2] Marian, M. et al. Exploring the lubrication mechanisms of synovial fluids for joint longevity–A perspective. *Colloids Surf., B*. **206**, 111926 (2021). 10.1016/j.colsurfb.2021.111926 34153619

[3] Rajankunte Mahadeshwara, M. et al. How do cartilage lubrication mechanisms fail in osteoarthritis? A comprehensive review. *Bioengineering* **11**, 541 (2024). 38927777 10.3390/bioengineering11060541 PMC11200606

[4] Sadique, M., Shah, S. R., Sharma, S. K. & Islam, S. M. Effect of significant parameters on squeeze film characteristics in pathological synovial joints. *Mathematics* **11**, 1468 (2023).

[5] Gonzales, G., Zauscher, S. & Varghese, S. Progress in the design and synthesis of viscosupplements for articular joint lubrication. *Curr. Opin. Colloid Interface Sci.* **66**, 101708 (2023).

[6] Morgan, D. W. & Welton, A. F. Inflammation Research Association proceedings of the sixth international conference. *Agents Actions* **39**, C3-C3 (1993).

[7] Sokolove, J. & Lepus, C. M. Role of inflammation in the pathogenesis of osteoarthritis: Latest findings and interpretations. *Therapeutic Adv. Musculoskelet. Disease*. **5**, 77–94 (2013). 10.1177/1759720X12467868 PMC3638313 23641259

[8] Mathiessen, A. & Conaghan, P. G. Synovitis in osteoarthritis: Current understanding with therapeutic implications. *Arthritis Res. Therapy*. **19**, 1–9 (2017). 10.1186/s13075-017-1229-9 PMC5289060 28148295

[9] Rutjes, A. W. et al. Viscosupplementation for osteoarthritis of the knee: A systematic review and meta-analysis. *Ann. Intern. Med.* **157**, 180–191 (2012). 22868835 10.7326/0003-4819-157-3-201208070-00473

[10] Rebenda, D. et al. Rheological and frictional analysis of viscosupplements towards improved lubrication of human joints. *Tribol. Int.* **160**, 107030 (2021).

[11] Peck, J. et al. A comprehensive review of viscosupplementation in osteoarthritis of the knee. *Orthoped. Rev.* **13** (2021). 10.52965/001c.25549 PMC8567800 34745480

[12] Grecomoro, G., Martorana, U. & Di Marco, C. Intra-articular treatment with sodium hyaluronate in gonarthrosis: A controlled clinical trial versus placebo. *Pharmatherapeutica* **5**, 137–141 (1987). 3310017

[13] Stitik, T. P. et al. Efficacy and safety of hyaluronan treatment in combination therapy with home exercise for knee osteoarthritis pain. *Arch. Phys. Med. Rehabil.* **88**, 135–141 (2007). 17270509 10.1016/j.apmr.2006.11.006

[14] Ishijima, M. et al. Intra-articular hyaluronic acid injection versus oral non-steroidal anti-inflammatory drug for the treatment of knee osteoarthritis: A multi-center, randomized, open-label, non-inferiority trial. *Arthritis Res. Therapy*. **16**, 1–8 (2014). 10.1186/ar4446 PMC3979073 24443804

[15] Adams, M. E. et al. The role of viscosupplementation with Hylan GF 20 (Synvisc^®^) in the treatment of osteoarthritis of the knee: A Canadian multicenter trial comparing Hylan GF 20 alone, Hylan GF 20 with non-steroidal anti-inflammatory drugs (NSAIDs) and NSAIDs alone. *Osteoarthr. Cartil.* **3**, 213–225 (1995). 10.1016/s1063-4584(05)80013-5 8689457

[16] Henrotin, Y. et al. Hyaluronan derivative HYMOVIS^®^ increases cartilage volume and type II collagen turnover in Osteoarthritic knee: Data from MOKHA study. *BMC Musculoskelet. Disord.* **20**, 1–16 (2019). 31215422 10.1186/s12891-019-2667-0 PMC6580647

[17] Vanelli, R., Costa, P., Rossi, S. M. P. & Benazzo, F. Efficacy of intra-articular polynucleotides in the treatment of knee osteoarthritis: A randomized, double-blind clinical trial. *Knee Surg. Sports Traumatol. Arthrosc.* **18**, 901–907 (2010). 20111953 10.1007/s00167-009-1039-y

[18] Kim, J. Y. et al. Pilot study to evaluate the efficacy of polynucleotide sodium compared to sodium hyaluronate and crosslinked sodium hyaluronate in patients with knee osteoarthritis. *J. Clin. Med.* **10**, 1138 (2021). 33803080 10.3390/jcm10051138 PMC7963169

[19] Dahl, L., Dahl, I., Engström-Laurent, A. & Granath, K. Concentration and molecular weight of sodium hyaluronate in synovial fluid from patients with rheumatoid arthritis and other arthropathies. *Ann. Rheum. Dis.* **44**, 817–822 (1985). 4083937 10.1136/ard.44.12.817 PMC1001790

[20] Marinho, A., Nunes, C. & Reis, S. Hyaluronic acid: A key ingredient in the therapy of inflammation. *Biomolecules* **11**, 1518 (2021). 34680150 10.3390/biom11101518 PMC8533685

[21] Kuppa, S. S. et al. Polynucleotides suppress inflammation and stimulate matrix synthesis in an in vitro Cell-based osteoarthritis model. *Int. J. Mol. Sci.* **24**, 12282 (2023). 37569659 10.3390/ijms241512282 PMC10418450

[22] Kim, J. G. et al. Safety and effectiveness of intra-articular injection of a highly cross-linked hyaluronic acid, LBSA0103 (Synovian): Results from a post-marketing surveillance study in South Korea. *Plos One*. **18**, e0287222 (2023). 37347765 10.1371/journal.pone.0287222 PMC10287010

[23] Ha, C. W. et al. Efficacy and safety of single injection of cross-linked sodium hyaluronate vs. three injections of high molecular weight sodium hyaluronate for osteoarthritis of the knee: A double-blind, randomized, multi-center, non-inferiority study. *BMC Musculoskelet. Disord.* **18**, 1–10 (2017). 28549436 10.1186/s12891-017-1591-4 PMC5446739

[24] Ebrahimpour, A., Bahrami, M., Raeissadat, S., Cheraghi, M. & Rahimi-Dehgolan, S. Efficacy of single high-molecular-weight versus triple low-molecular-weight hyaluronic acid intra-articular injection among knee osteoarthritis patients. (2020). 10.1186/s12891-020-03577-8 PMC7429877 32799851

[25] Kim, T. W., Chang, M. J., Shin, C. Y., Chang, C. B. & Kang S.-B. A randomized controlled trial for comparing efficacy and safety between intraarticular polynucleotide and hyaluronic acid for knee osteoarthritis treatment. *Sci. Rep.* **13**, 9419 (2023). 37296122 10.1038/s41598-023-35982-z PMC10256705

[26] Moon, J. Y. et al. Comparison of polynucleotide, sodium hyaluronate, and crosslinked sodium hyaluronate for the management of painful knee osteoarthritis: A multi-center, randomized, double-blind, parallel-group study. *Pain Med.* **24**, 496–506 (2023). 36255262 10.1093/pm/pnac155

[27] Choi, S. H. et al. Effects of a combination of polynucleotide and hyaluronic acid for treating osteoarthritis. *Int. J. Mol. Sci.* **25**, 1714 (2024). 38338992 10.3390/ijms25031714 PMC10855695

[28] Dallari, D. et al. Efficacy of intra-articular polynucleotides associated with hyaluronic acid versus hyaluronic acid alone in the treatment of knee osteoarthritis: A randomized, double-blind, controlled clinical trial. *Clin. J. Sport Med.* **30**, 1–7 (2020). 31855906 10.1097/JSM.0000000000000569

[29] Uygur, E., Türkmen, İ., Özturan, B. & Poyanli, O. Safety and efficacy of intra-articular 20 mg/2 ml hyaluronic acid injection for the non-operative palliation treatment of osteoarthritis of the knee joint. *Acta Chirurgiae Orthopaedicae Et Traumatologiae Čechoslovaca* 87 (2020). 32940223

[30] Becher, C., Springer, J., Feil, S., Cerulli, G. & Paessler, H. H. Intra-articular temperatures of the knee in sports–An in-vivo study of jogging and alpine skiing. *BMC Musculoskelet. Disord.* **9**, 1–7 (2008). 18405365 10.1186/1471-2474-9-46 PMC2330048

[31] Stagni, C. et al. Randomised, double-blind comparison of a fixed co-formulation of intra-articular polynucleotides and hyaluronic acid versus hyaluronic acid alone in the treatment of knee osteoarthritis: Two-year follow-up. *BMC Musculoskelet. Disord.* **22**, 1–12 (2021). 34511091 10.1186/s12891-021-04648-0 PMC8436495

[32] Hasnain, S. et al. Knee synovial fluid flow and heat transfer, a power law model. *Sci. Rep.* **13**, 18184 (2023). 37875531 10.1038/s41598-023-44482-z PMC10598223

[33] Cilurzo, F. et al. Injectability evaluation: An open issue. *Aaps Pharmscitech*. **12**, 604–609 (2011). 21553165 10.1208/s12249-011-9625-y PMC3134656

[34] Watt, R. P., Khatri, H. & Dibble, A. R. Injectability as a function of viscosity and dosing materials for subcutaneous administration. *Int. J. Pharm.* **554**, 376–386 (2019). 30414478 10.1016/j.ijpharm.2018.11.012

